# A cell engineering strategy to enhance supercoiled plasmid DNA production for gene therapy

**DOI:** 10.1002/bit.25971

**Published:** 2016-03-16

**Authors:** Sally Hassan, Eli Keshavarz‐Moore, John Ward

**Affiliations:** ^1^ The Advanced Center for Biochemical Engineering, Department of Biochemical EngineeringUniversity College LondonGordon StreetLondonWC1H 0AHUnited Kingdom

**Keywords:** plasmid DNA, supercoiling density, segregational stability, cell engineering, fermentation, *E. coli*

## Abstract

With the recent revival of the promise of plasmid DNA vectors in gene therapy, a novel synthetic biology approach was used to enhance the quantity, (yield), and quality of the plasmid DNA. Quality was measured by percentage supercoiling and supercoiling density, as well as improving segregational stability in fermentation. We examined the hypothesis that adding a Strong Gyrase binding Site (SGS) would increase DNA gyrase‐mediated plasmid supercoiling. SGS from three different replicons, (the Mu bacteriophage and two plasmids, pSC101 and pBR322) were inserted into the plasmid, pUC57. Different sizes of these variants were transformed into *E. coli* DH5α, and their supercoiling properties and segregational stability measured. A 36% increase in supercoiling density was found in pUC57‐SGS, but only when SGS was derived from the Mu phage and was the larger sized version of this fragment. These results were also confirmed at fermentation scale. Total percentage supercoiled monomer was maintained to 85–90%. A twofold increase in plasmid yield was also observed for pUC57‐SGS in comparison to pUC57. pUC57‐SGS displayed greater segregational stability than pUC57‐cer and pUC57, demonstrating a further potential advantage of the SGS site. These findings should augment the potential of plasmid DNA vectors in plasmid DNA manufacture. Biotechnol. Bioeng. 2016;113: 2064–2071. © 2016 The Authors. *Biotechnology and Bioengineering* Published by Wiley Periodicals, Inc.

Second to viral vectors, non‐viral, plasmid DNA currently accounts for over 17.7% of all gene therapy clinical trials (www.wiley.co.uk/genmed/clinical, 2015). The Federal Drug Administration (FDA) recommend that plasmids for therapy have a content of >80% in the supercoiled conformation (Morgan et al., 2002). This criterion is based on the view that supercoiled plasmid has a superior biological activity in comparison to other plasmid forms.

A high (>70%) supercoiled level requirement to elicit an effective immune response in cats and protection from infectious challenge with Rabies was previously demonstrated (Cupillard et al., 2005). Similarly, Pillai et al. (2008) showed that supercoiled DNA was three‐times more effective than open circular DNA at priming an MVA‐boosted CD8 T cell response in mice. In a separate study, the significance of supercoiled topology in the efficiency of transient transfection of CHO cells and dendritic cells was also demonstrated (Dhanoya et al., 2011, 2012).

DNA supercoiling refers to the degree of unwinding of a DNA duplex. A “relaxed” double‐helical segment of B‐DNA consists of two DNA strands twisting around the helical axis once every 10.4–10.5 sequence base pairs. The number of turns (*n*) in this linear DNA molecule would be its size in base pairs divided by 10.4 or 10.5. A relaxed circular DNA could be formed by joining the two ends of this molecule. In all known organisms with the exception of some hyperthermophilic bacteria, DNA supercoiling is negative that is, it possesses right‐handed superhelical turns. Bacterial growth medium conditions such as carbon to nitrogen ratio or culture time can be altered to increase the percentage of plasmid supercoiling in *E. coli* (O'Kennedy et al., 2000, 2003).

This article describes an original approach of increasing plasmid DNA supercoiled content by manipulating the plasmid itself. DNA supercoiling is tightly regulated in the cell by a homeostatic process, essential for cell viability. It is controlled by the enzymes DNA topoisomerase (which removes supercoils) and DNA gyrase (that adds supercoils) (Snoep et al., 2002). DNA gyrase binds to DNA at preferred sites (Morrison and Cozzarelli, 1981; Oram et al., 2003), cleaves the DNA duplex, passes another double‐stranded segment through the gap, re‐joins the cleaved DNA strands and uses the energy of ATP hydrolysis to release the DNA and complete the catalytic cycle, thereby introducing two negative supercoils. In a prior study, overexpression of DNA gyrase in bacterial strains led to only a very small increase in plasmid supercoiling (Snoep et al., 2002).

Initial research on gyrase binding to DNA indicated that gyrase bound with varying strength to binding sites on DNA molecules of about 140 bp. The plasmid ColEI, and viruses Mu, ϕX174 and SV40 all contain a range of binding sites (Morrison and Cozzarelli, 1981). Mu phage has a strong gyrase binding site (SGS) for DNA gyrase which forms an essential part of the bacteriophage's replication. Here we test the effect of the addition of SGS sequences from Mu phage, and from plasmids pBR322 (Lockshon and Morris, 1985) and pSC101 (Wahle and Kornberg, 1988), to the multicopy plasmid pUC57. Small and large versions of these fragments were investigated, since prior work has shown that DNA sequences either side of the central 140 bp of the SGS are important in its activity (Oram et al., 2003; Pato and Banerjee, 2000).

Three possible consequences of adding the strong gyrase binding site (SGS) were identified. Firstly, SGS may lead to an increase in the percentage of supercoiled pDNA relative to open‐circles. Alternatively or additionally, SGS may increase plasmid superhelical density, or increase the rate that plasmid molecules achieve their final superhelical density.

Supercoiling density was calculated according to Bowater et al. (1992). Figure 1 shows an example of a two‐dimensional chloroquine diphosphate gel used to analyze supercoiling density. For small‐scale studies carried out in falcon tubes, all analyses were performed on stationary phase cultures. Supercoiling density measurements for fermentations were taken at the start of the exponential phase to stationary phase. A plasmid preparation for pUC57‐SGS (Mu, 398) isolated from *E. coli* DH5α after an 8 h fermentation is shown in the gel in Figure 1. Linking number difference, ΔLk is determined by (Lk–Lk_0_), where Lk is linking number or number of superhelical turns for the supercoiled plasmid and Lk_0_ is linking number for the corresponding relaxed form of plasmid DNA. In order to determine the linking number difference, ΔLk, of each plasmid type from the two‐dimensional chloroquine diphosphate gels, all the topoisomers in the left‐hand arc, starting with the most relaxed form (ΔLk = 0) were counted. The supercoiling density or specific linking difference of the plasmid, α, was then determined by dividing ΔLk with Lk_0_, estimated by (*N*/h_0_), where *N* is the plasmid size in number of base pairs and h_0_ is the number of base pairs per turn of the DNA helix for circular B form of DNA in its relaxed state, known to be 10.5 bp/turn.

**Figure 1:**
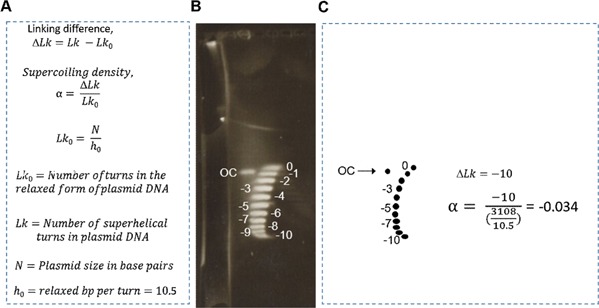
(**A**) Equations and definitions for calculating supercoiling density. (**B**) Plasmid preparations were run in the first dimension with 0.6 mg/L chloroquine diphosphate, and in the second dimension with 3 mg/L chloroquine diphosphate. The gel is showing an example plasmid preparation after an 8 h fermentation for pUC57‐SGS (Mu, 398) isolated from DH5α *E. coli* strain. (**C**) Counting method to determine ΔL_k_ in Figure 1B and supercoiling density, α, from ΔL_k_.

All seven types of pUC57‐SGS were prepared as synthetic genes in TOP10 strain of *E.coli* by GenScript Corporation (Piscataway, NJ). The resulting plasmids consisted of a mixture of dimers and monomers. Since, dimers and multimers would be considered as contaminants in the process, plasmids preparations isolated from the TOP10 strain of *E. coli* that were mainly monomer were propagated. The result of this was an average of 60% supercoiled monomer for all pUC57‐SGS regardless of SGS source (Appendix A). As not all dimers and multimers were completely eliminated in these plasmids, the plasmids were digested and re‐ligated before being transformed into a Rec^−^ strain of *E. coli* (that prevents plasmid multimerisation), DH5‐α. This was successful in achieving 100% monomer that consisted of more than 90% supercoiled plasmid DNA, with the remainder being open‐circles (Fig. 2A). This supports the findings in Yau et al. (2008) where, DH5‐α cells transformed with the 5.8 kb plasmid, gWiz had a similarly high supercoiled monomer content.

**Figure 2:**
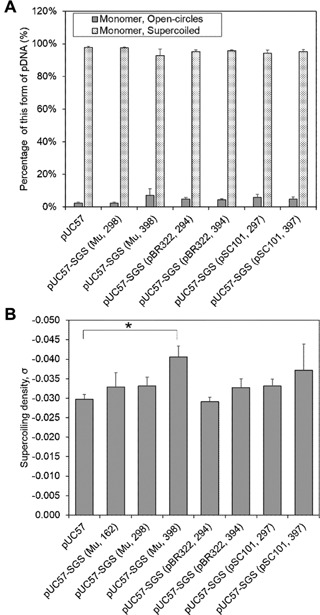
Supercoiling properties for plasmids pUC57 and pUC57‐SGS isolated from *E. coli* DH5‐α. Plasmid preparations were derived from overnight cell cultures of single colonies grown in 15 ml of LB media containing 100 μg/ml of ampicillin in a 50 ml falcon tube, at 37°C with horizontal shaking at 170 rpm. (**A**) Total percent supercoiling. Averages and SEMs are for 3, 3, 3, 4, 5, 4, and 5 separate plasmid DNA preparations, from left to right of the *x*‐axis. (**B**) Supercoiling density. Averages and SEM are for 3, 2, 3, 4, 3, 3, 2, and 2 replicates from left to right of the *x*‐axis. There was a statistically significant difference (*P* = 0.016) between pUC57 and pUC57‐SGS (Mu, 398), (*T*‐test, one tailed distribution, two samples equal variance, Excel 2013), as indicated by the asterisk above the graph.

Figure 2B shows that all pUC57‐SGS plasmids preparations in the *E. coli* host strain DH5α had the same supercoiling density as the control plasmid, pUC57, with the exception of pUC57‐SGS (Mu, 398) which showed a statistically significant 36% increase in supercoiling density relative to pUC57 (*P* = 0.016, *T*‐test, one‐tailed distribution, two‐samples equal variance, Excel 2013), with pUC57‐SGS (Mu, 398) possessing a linking number of about −12, unlike pUC57 with a linking number of about −8. These results indicate that the source and size of SGS are important for increasing plasmid supercoiling density.

No other sources of SGS led to a change in supercoiling density. This is most likely because the plasmid gyrase site, pBR322 is weaker than Mu SGS (Pato and Banerjee, 1999). On the other hand, pSC101 although it is a strong site that allows cleavage in the presence of gyrase, it is unable to promote Mu replication, indicating that it is a less efficient site (Pato and Banerjee, 1999). Further studies are required to establish whether this increase in supercoiling density improves plasmid integrity during downstream processing and uptake by cells for greater biological activity or efficiency, but studies demonstrating that percentage supercoiling (Cupillard et al., 2005; Dhanoya et al., 2011, 2012; Pillai et al., 2008) is important for uptake or activity, and for downstream processing (Li et al., 2011) indicate that this might be the case.

To show that the increases in superhelical density can be scaled, batch fermentations using bioreactors were performed. Growth profiles for pUC57 and pUC57‐SGS, were similar, reaching a maximal OD at 600 nm of about 20 (Fig. 3A). There was a steady increase in plasmid DNA yield with cell growth up to the maximal at 10–11 h of batch culture (Fig. 3B) and *E. coli* DH5α harboring plasmids with SGS were able to produce almost double the plasmid DNA yield of those harboring pUC57 alone with an average maximum of 9 μg/ml of culture. The reason for the increase in plasmid yield in DH5α Rec^−^ cells for pUC57‐SGS was not investigated further via experimentation, however, a possible reason is that in Rec^−^ strains, more efficient supercoiling causes more efficient DNA replication hence increasing plasmid yield. A higher superhelical density would potentially lead to easier melting of the DNA duplex and might enable the pRNA II promoter to initiate more often and/or facilitate the RNA/DNA duplex formation that is the precursor to the initiation of RNAse III cleavage and DNA replication.

**Figure 3:**
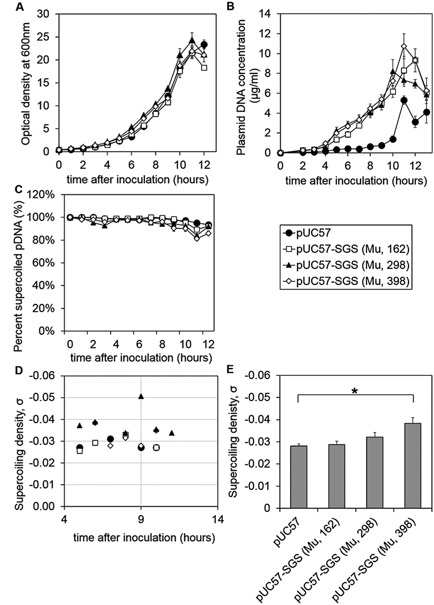
Batch fermentation of plasmids pUC57 and pUC57‐SGS (with SGS derived from Mu, for 162, 298, and 398 bp of the SGS) in DH5α *E. coli* cells. Error bars represent standard error of the mean, (SEM). (**A**) Optical density at 600 nm. (**B**) DNA concentration per ml of culture. Plasmid DNA measurements were the result of Qiagen Minipreps on triplicate cell culture samples. (**C**) Total percent supercoiling. (**D**) Supercoiling density with culture time. Supercoiling density results are shown for three different fermentations performed under the same conditions. (**E**) Average of supercoiling density shown in D. There was a statistically significant difference (*P* = 0.008) between pUC57 and pUC57‐SGS (Mu, 398), (*T*‐test, one tailed distribution, two samples equal variance, Excel 2013), as indicated by the asterisk above the graph.

Percentage supercoiled monomer was successfully maintained at about 90% from the beginning of the fermentation to mid‐exponential phase in all cases, dropping only slightly to about 85% from mid‐exponential phase to the end of the fermentation (Fig. 3C), possibly due to greater energy used by the cells at this later point in the batch fermentation, in cell division and in plasmid DNA replication. Figure 3D and E show that, similarly to the small‐scale results in 15 ml cultures grown under less controlled conditions, for pUC57‐SGS (Mu, 398) grown in this *E. coli* strain, there was significant, 36%, increase in supercoiling density relative to the parent plasmid, pUC57, (*P* = 0.008, *T*‐test, one‐tailed distribution, two‐samples equal variance, Excel 2013). A prior study (Yau et al., 2008) however, has shown that percentage supercoiling is affected by *E. coli* host strain and our studies have shown that benefits of the SGS (Mu, 398) are not visible in strains allowing plasmid multimerisation (in BL21 DE3 cells, Appendix B), and hence careful choice of *E. coli* host strain for plasmid DNA production is vital.

Another important consideration for plasmid DNA manufacture is the current presence of antibiotic in the medium, which is not favored by the regulatory authorities for gene therapy or DNA vaccination due to the possibility of horizontal transfer of antibiotic resistance to the circulating microbial population (Sodoyer et al., 2012). Antibiotics are traditionally added to kill plasmid‐free cells that arise due to the segregational instability of the plasmid replicons, thus increasing the segregational stability of plasmids could be beneficial in enabling growth in the absence of antibiotics.

Most multicopy plasmids used in molecular biology and gene therapy are derivatives of the Col EI replicon (Prather et al., 2003). This plasmid is naturally occurring, highly stable and gives rise to no plasmid‐free cells during replication and cell division. However, during the process of creating the most common high copy number vectors, several DNA regions have been removed. One of these DNA sections, *cer*, contains a binding site for the recombinase XerC. The recombinase enzyme handles reversing dimer and higher multimer formation by recombination across two *cer* sites on the same molecule thus maintaining Col EI replicons as monomers. Plasmid multimer formation is segregationally unstable and leads to “dimer catastrophe” (Summers et al., 1993) and segregation of plasmid‐free cells. French and Ward (1995) demonstrated that the insertion of a 150 bp DNA fragment containing *cer* can confer segregational stability to a plasmid such that no antibiotic is needed in fermentation and 100% of the cells can successfully retain plasmids. The addition of the *cer* fragment to the multicopy plasmid, pUC57 should prevent mutimer formation and increase the segregational stability of these plasmids. In this article, we use synthetic biology to increase the level of plasmid supercoiling and plasmid segregational stability, to build an improved backbone vector for plasmid DNA bioprocessing.

Plasmid segregational stability was analyzed as described in French and Ward (1995). Figure 4 shows the percentage plasmid retaining cells with increasing numbers of bacterial transfer into fresh media and growth in the absence of antibiotics for 24 h at 37°C for pUC57, pUC57‐*cer*, pUC57‐SGS (Mu, 298), pUC57‐SGS (Mu, 298)‐*cer*, pUC57‐SGS (pBR322, 294), and pUC57‐SGS (pSC101, 297).

**Figure 4:**
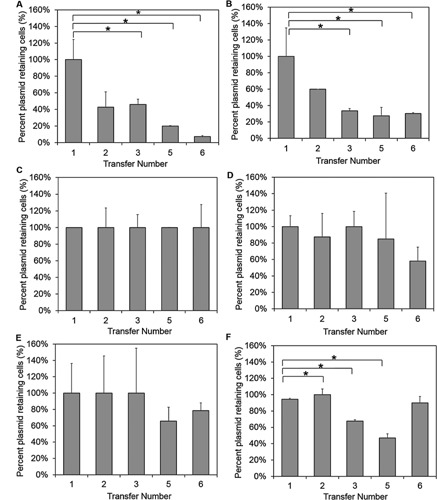
Plasmid segregational stability. Percentage of plasmid retaining cells with increasing numbers of bacterial transfer into fresh media in the absence of antibiotics for 24 h at 37°C for (**A**) pUC57, (**B**) pUC57‐*cer*, (**C**) pUC57‐SGS (Mu, 298), (**D**) pUC57‐SGS (Mu, 298)‐*cer*, (**E**) pUC57‐SGS (pBR322, 294), and (**F**) pUC57‐SGS (pSC101, 297). Averages and SEMs are shown for duplicates. Statistically significant decreases (*P* < 0.05, *T*‐test, one tailed distribution, two samples equal variance, Excel 2013) between the first transfer and subsequent transfers are indicated by an asterisk above the plots.

Figure 4A and B shows that the *cer* fragment, can partially improve segregational plasmid stability when added to pUC57 and analyzed in the DH5α strain of *E. coli*, but this construct drops to 30% of plasmid containing cells and stays there for three non‐selective growths (Fig. 4B). pUC57‐SGS in DH5α displayed even greater segregational stability (Fig. 4C–F). This increase was highest for pUC57‐SGS with the Mu, (298) fragment where 100% of the cells retained the plasmid through six cycles of non‐selective growth (Fig. 4C). Although the further addition of a *cer* site to this plasmid (Fig. 4D) seemed to indicate a decrease in plasmid segregational stability of the plasmid with only 55% of cells ultimately retaining the plasmid, there was no statistically significant difference between this and the first transfer (*P* = 0.065, *T*‐test, one‐tailed, two samples equal variance, Excel 2013).

In hindsight, it would have been better to test puC57‐SGS (Mu, 398) with cer rather than pUC57‐SGS (Mu, 298) as this showed the best supercoiling density, however, this experiment was conducted to test whether segregational stability could be increased using the cer fragment, regardless of whether there was an SGS site present. To this effect, pUC57‐SGS (Mu, 298), and pUC57 alone were tested, with the addition of a cer site. Although it was an expected result that cer would increase segregational stability, we did not expect the SGS site also to increase plasmid segregational stability. After the results with pUC57‐SGS (Mu, 298), we also tested pUC57‐SGS (pBR322, 294) and pUC57‐SGS (pSC101, 297) to see whether, the benefits of SGS on segregational stability were specific to the source of the SGS site. Both pUC57‐SGS (pBR322, 294) and pUC57‐SGS (pSC101, 297) increased segregational stability of the DH5‐α *E. coli* cells to a level where on average 70% of the cells retained the plasmid (Fig. 4E and F), with no statistical difference between the first and last transfers for the former but a statistically significant drop between the first and second transfers in the latter (*P* = 0.003). This indicates that in Rec^−^ strains of *E. coli*, the SGS has an unforeseen benefit of improving the segregational stability of the plasmids, demonstrating a further advantage of this site. The increase in plasmid segregational stability with the strong gyrase binding site may be due to having gyrase bound and/or an increased superhelical density; however, a mechanism for how this leads to an increase in segregational stability awaits further study.

These results demonstrate that alongside the conventional methods of optimizing fermentation conditions, synthetic biology can be used to optimize plasmid DNA yield, supercoiling density, and segregational stability. SGS (∼296 bp) sourced from the Mu phage, and plasmids pBR322 and pSC101 was found to enhance plasmid segregational stability. Mu phage‐sourced SGS was also found to enhance pUC57 yield, and the larger fragment of this site (398 bp) increased supercoiling density. Further work is required to demonstrate whether increased plasmid supercoiling density by the addition of this SGS site leads to the anticipated increase in transfection efficiency. Nonetheless, applying supercoiling density measurements together with traditional percentage supercoiling analysis could be a useful analytical tool or product characterization method in the plasmid DNA production process.

## Materials and Methods

### Construction of Plasmids

The strong gyrase binding sites (SGS) from three different replicons were designed and ordered as synthetic genes from GenScript Corporation. The regions of SGS from each of these replicons was designed based on the data in Oram et al. (2003) and were reduced or extended for desired lengths from the sequences of Mu (Morgan et al., 2002), pBR322 (Lockshon and Morris, 1985), and pSC101 (Wahle and Kornberg, 1988). Three lengths of the Mu fragment (162, 298, and 398 bp) and two fragments of pBR322 (294 and 394 bp) and pSC101 (297 and 397 bp) were obtained. These fragments were inserted into the XbaI site in the 2.7 kb plasmid, pUC57 by GenScript Corporation.

The *cer* sequence from ColE1, described by Summers and Sherratt (1988), Summers (1989), and Balding et al. (2006), was extended beyond its Rcd transcript and was designed to have restriction sites of KasI and PfoI at both ends. This 309 bp sequence was ordered as a synthetic gene from Eurofins MWG operon, (Ebersberg, Germany) and was then inserted into the PfoI site of pUC57 and pUC57‐SGS (Mu, 298) and transformed into *E.coli* DH5α using standard cloning methods (Sambrook and Russell, 2001).

### Culture and Plasmid Isolation for 15 ml, Bench Scale Experiments

A single colony from DH5α *E. coli* transformed cells was grown overnight at 37°C in 15 ml Luria‐Bertani (LB) media with 100 μg/ml Ampicillin. Overnight culture was pelleted and the plasmid constructs isolated using standard protocols in the QIAprep miniprep kit (Qiagen, Crawley), with elution in 30 μl of elution buffer.

### Parallel Fermentation in Semi‐Defined Media

Fermentations were performed using four parallel 1 L fermenters, Multifors, (Infors‐HT, Bottmingen, Switzerland), using 700 ml of the semi‐defined medium containing casamino acids, SDCAS (O'Kennedy et al., 2000), with 100 μg/ml Ampicillin, and inoculated with 20 h shake flask culture, so that the starting OD at 600 nm was 0.4. To prepare the inoculum, a single colony was grown in 5 ml of Luria‐Bertani (LB) containing 100 μg/ml ampicillin for 20 h and this was used to inoculate 100 ml of fresh LB media containing ampicillin in a 250 ml shake flask for a further 20 h. Fifty milliliters of the second inoculum was then used to inoculate 100 ml of SDCAS medium at pH 7 for 20 h, and this was used to inoculate the fermenters as described above. Fermentation conditions were maintained at pH 7 and 37°C and air flow of 1.4 vvm, with dissolved oxygen tension being maintained at 30% or above with a stirrer speed of 500–1,100 rpm. A few drops of 100% polyethylene glycol (PEG) were added as required, to control foaming at the surface of the culture.

### Agarose Gel Electrophoresis for Percentage Supercoiling Determination

This was determined as described by Yau et al. (2008) by comparing the percentage of open‐circular bands with supercoiled bands using densitometer scanning of the agarose gels.

### Two‐Dimensional Chloroquine Diphosphate Agarose Gels for Supercoiling Density Determination

Two samples were loaded 8–10 cm apart and electrophoresed in the first dimension at a chloroquine diphosphate concentration of 0.6 mg/L, at 10 V and 24–26 mA for 17 h. The gel was then allowed soak for equilibration in the second electrophoresis buffer (3 mg/L chloroquine diphosphate in 1XTBE) for 3 h. Electrophoresis in the second dimension was performed at 90° to the first dimension at 10 V and 24–26 mA for 20.5 h.

Gels were then rinsed thrice in 1× TBE for an hour each, and were then stained with SYBR Gold nucleic acid gel stain (Molecular Probes, Invitrogen, Paisley, UK) according to the manufacturer's instructions for 2 h before visualisation under UV light. Gels were scanned with Gene Snap version 7.07.01 (Syngene, Synoptics Ltd., Cambridge, UK). Supercoiling density was determined according to Bowater et al. (1992). For small‐scale experiments in 50 ml tubes, average supercoiling density was a result of at least three separate plasmid preparations.

### Plasmid Segregational Stability

This was carried out as described by French and Ward (1995). In brief, glycerol stocks from DH5α pUC57, pUC57‐*cer*, pUC57‐SGS (Mu, 298), pUC57‐SGS Mu, (298)‐*cer*, DH5α pUC57‐SGS (pBR322) and pUC57‐SGS (pSC101) were streaked out onto selective LB agar plates (containing 100 μg/ml ampicillin) and incubated at 37°C for 16 h. A single colony from each was subsequently inoculated into 50 ml of non‐selective LB media and incubated at 37°C, 200 rpm for 24 h. The resulting stationary phase culture was then diluted 10^6^‐fold into fresh LB without antibiotics and grown for a further 24 h. This process of dilution and non‐selective growth was repeated for 5 days. Serial dilutions were made from each 24 h culture just before dilution for the repeated growth, and plated out as duplicates onto selective and non‐selective agar plates. The percentage of plasmid retaining cells was determined from the ratio of colonies on selective plates over those on non‐selective plates.

We are grateful to the BBSRC and the Bioprocessing Research Industry Club (BRIC) for funding this project. BBSRC grant BBE0060191.

## Appendix

Percentage supercoiling for pUC57 and the various pUC57‐SGS isolated from TOP10 strain of *E.coli*. Averages and SEMs are of 3, 13, 8, 13, 15, 9, 13, and 12 separate plasmid DNA preparations from left to right on the *x*‐axis. All plasmid preparations were derived from a 15 ml overnight cell culture in a 50 ml falcon tube of a single colony in LB media containing 100 μg/ml of ampicillin, at 37°C with horizontal shaking at 170 rpm.

## Appendix

Plasmid supercoiling density of pUC57 and pUC57‐SGS in BL21 DE3 *E.coli* cells following a 7 h fermentation. BL21 DE3 are Rec^+^ cells that were unable to prevent plasmid multimerisation and hence plasmid dimer and multimers were also present. Supercoiling density measurements are only shown for plasmid monomers, with no notable difference between the groups. Averages and SEMs are of duplicates and triplicates.
